# Prognostic Significance of Serum Alkaline Phosphatase Level in Osteosarcoma: A Meta-Analysis of Published Data

**DOI:** 10.1155/2015/160835

**Published:** 2015-11-04

**Authors:** Hai-Yong Ren, Ling-Ling Sun, Heng-Yuan Li, Zhao-Ming Ye

**Affiliations:** Department of Orthopaedics, Second Affiliated Hospital, College of Medicine, Zhejiang University, No. 88, Jiefang Road, Hangzhou 310009, China

## Abstract

*Background*. Serum alkaline phosphatase (SALP) is commonly elevated in osteosarcoma patients. A number of studies have investigated the prognostic role of SALP level in patients with osteosarcoma but yielded inconsistent results. *Method*. Systematic computerized searches were performed in PubMed, Embase, and Web of Science databases for relevant original articles. The pooled hazard ratios (HRs) and relative risks (RRs) with corresponding confidence intervals (CIs) were calculated to assess the prognostic value of SALP level. *Results*. Finally, 21 studies comprising 3228 patients were included. Overall, the pooled HRs of SALP suggested that elevated level had an unfavorable impact on osteosarcoma patients' overall survival (OS) (HR = 1.82; 95% CI: 1.61–2.06; *p* < 0.001) and event-free survival (EFS) (HR = 1.97; 95% CI: 1.61–2.42; *p* < 0.001). Combined RRs of SALP indicated that elevated level was associated with presence of metastasis at diagnosis (RR = 5.55; 95% CI: 1.61–9.49; *p* = 0.006). No significantly different results were obtained after stratified by variables of age range, cancer stage, sample size, and geographic region. *Conclusion*. This meta-analysis demonstrated that high SALP level is significantly associated with poor OS or EFS rate and presence of metastasis at diagnosis. SALP level is a convenient and effective biomarker of prognosis for osteosarcoma.

## 1. Introduction

Osteosarcoma is the most common primary bone tumor in childhood and adolescence. It is the second highest cause of cancer-related death in these age groups due to development of often fatal metastasis, usually in the lungs [1]. Prior to the use of chemotherapy, 80–90% of patients with osteosarcoma developed metastatic disease, despite achieving local tumor control, and died of their diseases [2]. Although multidisciplinary management including neoadjuvant and adjuvant chemotherapy with aggressive surgical resection has improved clinical outcomes, the overall 5-year survival rate remains 60–70% [3]; the treatment of osteosarcoma is still unsatisfactory for the risk of local relapse and the development of metastasis [4, 5]. Osteosarcoma has a predilection for metastasizing to the lungs. Pulmonary metastases occur in approximately half of the osteosarcoma patients and are the main cause of death for patients with osteosarcoma. At the time of osteosarcoma diagnosis, fewer than 20% of patients present with identified metastatic diseases [6–8], while most patients with localized osteosarcoma are assumed to have undetectable micrometastases [9–12]. The 5-year OS rate for patients with metastatic spread is less than 30%, largely unchanged during the past 30 years [8, 13]. Therefore, valuable prognostic factors should be found to identify the high-risk patients efficiently, and aggressive therapeutic regimens could be initiated earlier on these patients to improve prognosis.

Alkaline phosphatases (ALPs) are a family of metalloenzymes that catalyze the hydrolysis of organic phosphate esters at an alkaline environment with low substrate specificity [14]. Four genes encode ALP, including tissue-nonspecific ALP (TNAP) gene located on 1p36.12, which is expressed in various tissues such as osteoblasts, hepatocytes, kidney, and early placenta, and three tissue-specific ALP genes located on 2q37, which are expressed in intestine (IAP), placenta (PLAP), and germ cells (placental-like AP or GCAP), respectively [15]. In healthy individuals, SALP derives mostly from bone, hepatic tissues, or kidney [16]. It is known that patients with osteosarcoma are commonly detected with increased SALP levels. The relationship between total SALP activity and clinical outcome of osteosarcoma patient has been recognized for over 50 years [17]. However, studies on the prognostic role of SALP level with osteosarcoma have yielded inconsistent results. Thus, we conducted a meta-analysis of all available studies relating SALP with survival rate or metastasis to clarify its prognostic value. In addition, normal value of SALP is complicated in children and adolescents, SALP is usually greater in children than in adults [18], which would confound its prognostic role on osteosarcoma patients. Cancer stage and other factors might also influence the results. Thus, the stratified analyses were further conducted to explore any difference in each subgroup.

## 2. Methods

### 2.1. Search Strategy and Selection Criteria

We searched PubMed, Embase, and Web of Science databases on May 1, 2015, for relevant articles. The search terms were used as follows: (1) osteosarcoma or bone sarcoma or osteogenic sarcoma and (2) alkaline phosphatase or ALP or SALP or SAP or AP or AKP or ALKP. Studies were considered eligible if they met the following criteria: (1) prospective or retrospective cohort study; (2) tumors being histologically confirmed as osteosarcoma; (3) studies examining the relation between SALP level and prognosis (OS, EFS, or metastasis); (4) publications written in English; (5) studies providing sufficient information to estimate HR or RR with corresponding 95% CIs. The exclusion criteria included (1) articles published in non-English; (2) case reports, editorials, letters, reviews, and conference abstracts; (3) only the most recent or complete study, when multiple publications from a particular research group reported data from overlapping samples.

### 2.2. Data Extraction and Study Assessment

Two reviewers extracted data from eligible studies independently. Discontents between reviewers were resolved by discussion and through consultation. The following items were collected from each study: first author's name, year of publication, country, sample size, age, cut-off values, tumor stage (Enneking stage), follow-up time, HRs of the elevated SALP for OS or EFS, RRs of the elevated SALP and presence of metastasis at diagnosis or metastasis development of localized osteosarcoma patients, and their 95% CIs and *p* values and other relevant data. Methodological quality of the included studies was assessed with the Newcastle-Ottawa Scale (NOS) [19].

### 2.3. Statistical Analysis

For each individual study with assessment of OS or EFS, the HRs and their 95% CIs were extracted if the author had reported the data. Otherwise, these data were calculated according to the methods described by Parmar et al. [20]. RRs with corresponding 95% CIs were used to measure the relationship of SALP level and presence of metastasis at diagnosis or metastasis development of localized osteosarcoma patients. Subgroup analyses were then conducted according to clinical variables including age range, tumor stage, sample size, and geographic region. Heterogeneity between the studies was measured by *Q* test and *I* ^2^ test [21, 22], while potential publication bias was investigated using funnel plot and Begg's test [23]. The fixed effects model was employed to combine the individual HR or RR estimates when there was no significant heterogeneity among studies; otherwise, the random effects' model was used [24]. Finally, sensitivity analysis was performed to assess the influence of the single study on the combined HR of OS or EFS. All statistical analyses were conducted using STATA 12.0 software (Stata Corporation, College Station, Texas, U.S.).

## 3. Results

### 3.1. Study Characteristics and Quality Assessment

**Figure 1:**
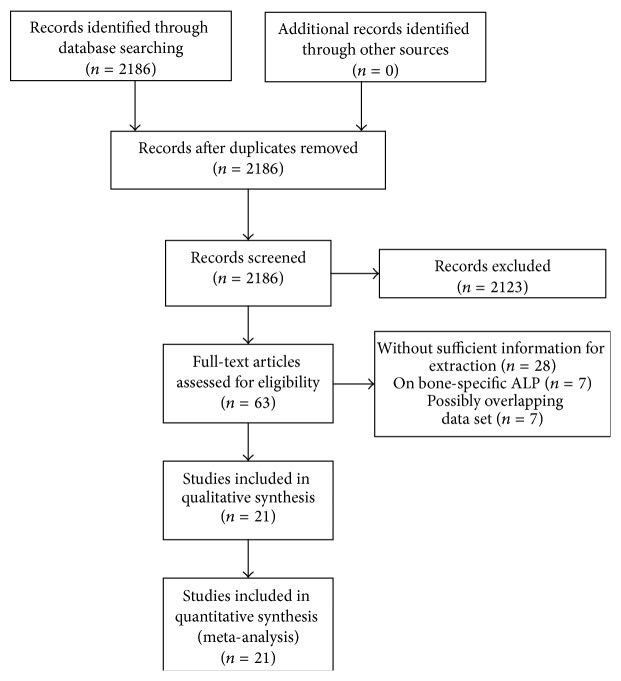
Flow diagram of the study selection process.

2186 relevant citations were identified for initial review using search strategies as described previously. Of these, 2123 were initially excluded after reading the titles and abstracts and 42 were excluded after assessing the full texts (28 studies without sufficient information for extraction, 7 studies on bone-specific ALP, and 7 studies by same authors on possibly the same patient populations) (Figure 1). Ultimately, the systematic literature search yielded a total of 21 studies comprising 3228 patients for final analyses [25–45]. These studies were conducted in nine countries and published between 1993 and 2015, each including patients ranging from 33 to 350 (median 91). The major characteristics of the 21 eligible publications are reported in Tables 1–4, each with studies on analyses of OS, EFS, presence of metastasis at diagnosis, and metastasis development among nonmetastatic patients, respectively.

HRs of OS could be extracted from 17 studies (Table 1) [25–41] and of EFS could be extracted from 7 studies (Table 2) [29–32, 36, 41, 42], respectively. Three of the included studies investigated the association between SALP level and presence of metastasis at diagnosis (Table 3) [33, 40, 43]. Other 3 studies recruited nonmetastatic patients and investigated the correlation between SALP level and risk of metastasis development (Table 4) [37, 44, 45]. Quality assessments revealed average NOS score from the two reviewers of 6.86, indicating that all 21 included studies were of moderate quality.

### 3.2. SALP Level and EFS or OS

**Figure 2:**
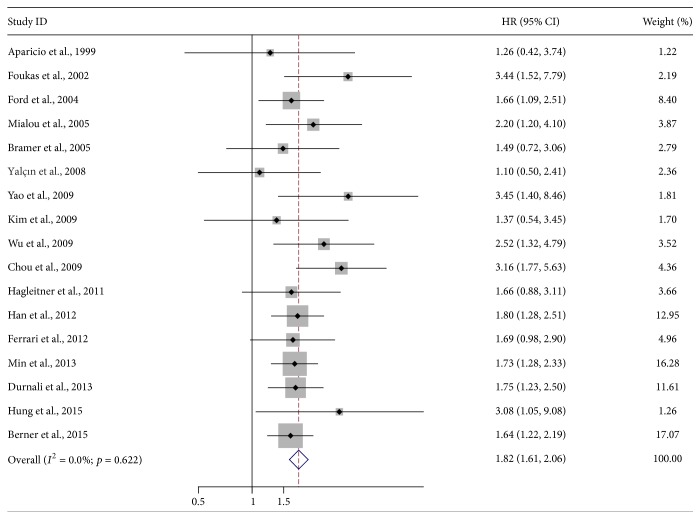
Forest plot showing the association between SALP and overall survival (OS) of osteosarcoma.

**Figure 3:**
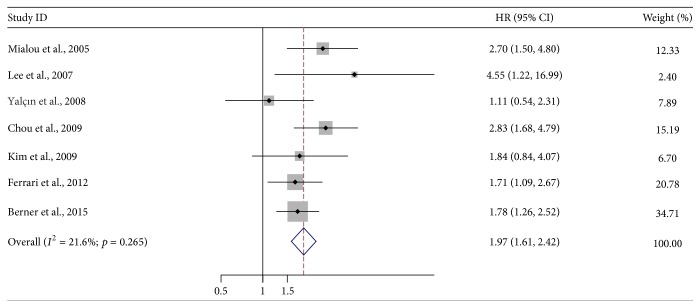
Forest plot showing the association between SALP and event-free survival (EFS) of osteosarcoma.

17 studies with a total of 2272 osteosarcoma patients dealing with SALP level and OS were meta-analyzed [25–41]. Because of heterogeneity (*I* ^2^ = 0%), a fixed effect model was selected. The pooled HR was 1.82 (95% CI: 1.61–2.06; *Z* = 9.73; *p* < 0.001), illustrating that SALP level was significantly associated with the poor OS of osteosarcoma patients (Figure 2). Seven studies including 752 patients which reported the correlation between SALP level and EFS were also meta-analyzed [29–32, 36, 41, 42]. No heterogeneity was detected (*I* ^2^ = 21.6%), so a fixed effect model was adopted. The combined HR was 1.97 (95% CI: 1.61–2.42; *Z* = 6.50; *p* < 0.001), demonstrating that SALP level of osteosarcoma patients was significantly associated with poor EFS (Figure 3).

### 3.3. SALP Level and Metastasis

**Figure 4:**
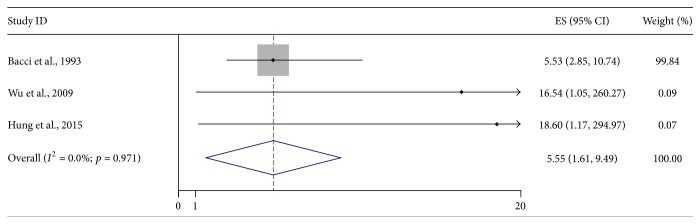
Forest plot showing the association between SALP and presence of metastasis of osteosarcoma at diagnosis.

**Figure 5:**
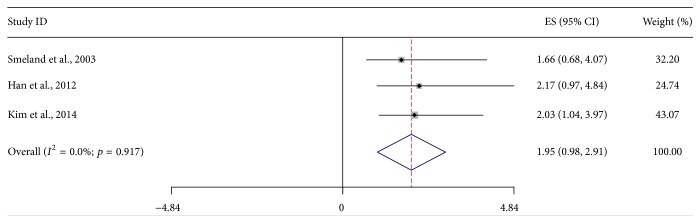
Forest plot showing the association between SALP and occurrence of metastasis for nonmetastatic osteosarcoma patients.

Three studies with 816 patients investigated the relationship between SALP level and presence of metastasis at diagnosis [33, 40, 43]. A fixed effect model was employed for analysis since no heterogeneity was detected (*I* ^2^ = 0.0%). The combined RR was 5.55 (95% CI: 1.61–9.49; *Z* = 2.76; *p* = 0.006), indicating significant relationship between elevated SALP level and metastatic disease of osteosarcoma patients (Figure 4). Moreover, other 3 studies, including 372 nonmetastatic osteosarcoma patients, observed the linkage of SALP level and metastasis development [37, 44, 45]. Because of heterogeneity (*I* ^2^ = 0%), a fixed effect model was used in this analysis. However, the result showed no statistically significant correlation between high SALP and metastasis development, with RR being 1.95 (95% CI: 0.98–2.91; *Z* = 3.96; *p* < 0.001) (Figure 5).

### 3.4. Subgroup Analyses

Because of the limited articles about metastasis, stratifying analysis was only conducted on the correlation between SALP and OS or EFS. Main results of subgroup analysis for OS and EFS were listed in Tables 5 and 6. After stratified by age range, the pooled HRs of preadult group (patients' age less than 19 years old) of OS and EFS were 1.87 (95% CI: 1.20–2.91; *Z* = 2.78; *p* = 0.242) and 2.07 (95% CI: 1.51–2.83; *Z* = 4.56; *p* = 0.341), respectively, similar to the studies comprising both preadult and adult patients, of which the HRs of OS and EFS were 1.82 (95% CI: 1.60–2.06; *Z* = 9.33; *p* = 0.625) and 1.90 (95% CI: 1.45–2.48; *Z* = 4.66; *p* = 0.149), respectively. When stratified by cancer stage, the association between SALP levels and prognosis among osteosarcoma patients seemed to be strengthened in the subgroup of metastatic patients (Enneking stage III), with HRs of OS and EFS being 2.67 (95% CI: 1.75–4.06; *Z* = 4.56; *p* = 0.400) and 2.77 (95% CI: 1.88–4.09; *Z* = 5.13; *p* = 0.906), respectively, while in the subgroup of localized osteosarcoma patients (Enneking stage II), the HRs of OS and EFS were 1.75 (95% CI: 1.42–2.15; *Z* = 5.29;  *p* = 0.740) and 1.88 (95% CI: 1.29–2.73; *Z* = 3.31; *p* = 0.386), respectively. Stratified analysis according to sample size was also conducted. Whether patient number is greater than 100 or not, similar results were found about the association between SALP level and poor OS or EFS, while apparent less heterogeneity was obtained in studies with larger sample size (*p* = 0.999; *I* ^2^ = 0.0%). When stratifying by geographic region, the HRs of high SALP for OS and EFS were not significantly different between subgroups of Asia or non-Asia.

### 3.5. Publication Bias and Sensitivity Analysis

**Figure 6:**
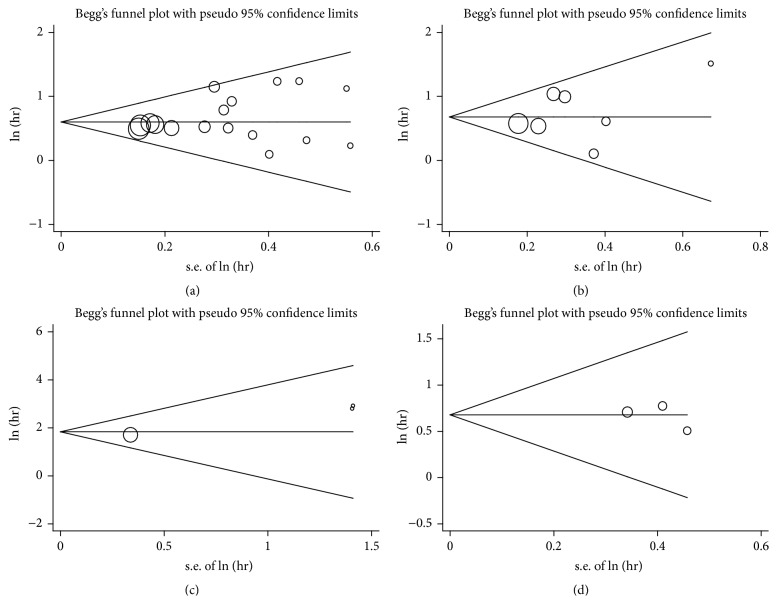
Funnel plots assessing possible publication bias for prognosis ((a) OS; (b) EFS; (c) presence of metastasis at diagnosis; (d) metastasis development for nonmetastatic patients).

**Figure 7:**
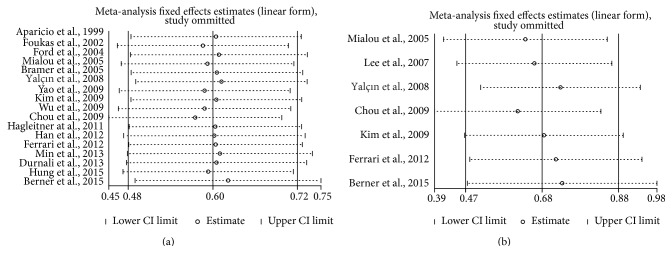
Sensitivity analysis for prognosis of survival rates ((a) OS; (b) EFS).

Publication bias of the included studies was assessed by funnel plots and Begg's test. As shown in Figure 6, the funnel plots were almost symmetric in each analysis. Meanwhile, one study was omitted at a time in the sensitivity analysis to measure its effect on the pooled HR for the OS or EFS. No individual study dominantly influenced overall HR, as presented in Figure 7, indicating the robustness of the results in this meta-analysis.

## 4. Conclusion

In the early studies, elevated SALP levels had been reported in 40% to 80% of patients with osteosarcoma [46–49]. In accordance with that ratio, of these selected studies which have sample size larger than 100 [27, 36–39, 41, 43, 44], elevated SALP levels were found in 40.2% to 83.7% of osteosarcoma patients. The relationship of serum total ALP activity with clinical outcomes of osteosarcoma has been recognized for over 50 years [17]; however, this remains controversial. Thus, to derive a more precise estimation of the correlation between SALP levels and survival rates or metastasis in patients with osteosarcoma, we carried out this meta-analysis.

The present meta-analysis suggested that osteosarcoma patients with high SALP levels have significantly poorer OS or EFS when compared with those with normal levels. The results also showed that patients with high SALP significantly correlated with greater ratio of presence of metastasis at diagnosis, indicating that osteosarcoma metastases obviously relate to higher SALP levels. However, it failed to obtain significant correlation between SALP level and metastasis development through nonmetastatic osteosarcoma patients, with the combined RR being 1.95 (95% CI: 0.98–2.91). Among the included three studies, it is worthwhile to notice that Kim et al. [45] developed a high-performance nomogram with several predictors to predict the probability of metastasis, including the factor of SALP level. Though the meta-analysis showed no statistically significant result, some relevance might exist between SALP level and metastasis development in localized osteosarcoma patients. Merely three studies were included in our analysis and more researches are needed to provide solid data to clarify this relation.

Assessment of SALP levels in children and adolescents is difficult because those levels are usually greater than in adults, they show a tetrabasic pattern with the highest levels in infancy and puberty and troughs at mid-childhood and at the end of puberty [18, 50, 51]. Normal value of SALP is complicated in preadult osteosarcoma patients; variant normal cut-off values were found through the studies, which would confound the results. The results of subgroup analyses revealed similar prognostic role of SALP between adult and preadult patients. However, more efforts are required to refine the prognostic value of SALP by different age, especially among preadult patients. Metastasis is the most crucial prognostic factor in osteosarcoma; patients with localized or metastatic osteosarcoma might have apparently different results. The analyses indicated that the association between SALP levels and survival outcomes were both significant for patients whether with metastasis or not, while the predictive value appears to be stronger among those patients with metastasis. Elevated SALP might tend to predict clinical outcomes more efficiently among metastatic osteosarcoma patients. No significantly different results were found after stratified by variables of sample size and geographic region.

In previous studies, cultured human osteosarcoma cell lines [52, 53] and an animal osteosarcoma cell line [54] have been shown to produce large amount of ALP. It is suggested that transformed osteoblasts in osteosarcoma would disrupt the tight control of proliferation and progressively express the genes associated with cell differentiation, causing a constantly high level of ALP [55, 56]. It is reported that SALP levels were significantly increased in osteoblastic subtype of osteosarcoma than in other subtypes [37]. In addition, osteosarcoma metastasis is associated with expansion and infiltration of tumor cells, stimulating local secretion of cytokines or growth factors and causing the activation of osteoclasts, which aggravate the severity of osteolysis accompanying SALP elevation [57]. The study of Han et al. [37] indicated that the matrix metalloproteinases (MMPs) can be secreted by the cancer cells to dissolve extracellular matrix, which may also lead to rise of SALP. It is reasonable to assume that osteosarcoma progression, invasion, or metastasis would aggravate osteolysis and elevate SALP. High level of SALP would relate to propensity for malignancy of osteosarcoma and poor clinical outcomes. It is reported that, in most patients with initial elevated SALP, the values decreased to be normal after preoperative chemotherapy [28, 37, 43]. Other than the SALP levels at diagnosis, posttreatment SALP values also gain great attention by researchers for its prognostic role. The Rizzoli Institute [43] analyzed SALP value after neoadjuvant chemotherapy and surgery in patients with initial high levels of the enzyme but failed to find significant relationship with relapse. However, Bramer et al. [28] and Han et al. [37] indicated that elevated postchemotherapy SALP correlated with shorter survival and greater incidence of lung metastasis as well as poor response to chemotherapy, though the correlations with relapse were also not evident in these two studies. Decrease of SALP level during clinical therapy may be a symptom of a positive reaction to treatment and disease remission. SALP which remains elevated after treatment might indicate unfavorable treatment response and predict poor prognosis.

Meanwhile, some limitations in this meta-analysis should be noticed. First, publication bias might be present if studies unpublished or in other languages that meet the inclusion criteria were missed. The tendency to publish positive findings over negative results may also introduce some bias. Second, some studies included both children and adults in one group, and the effect of age on the levels of SALP was not taken into account for their analyses. Moreover, normal value of SALP is complicated in preadult osteosarcoma patients; most of the studies including young patients did not apply corresponding cut-off point detailed enough by age, which would make the results less accurate. Third, HRs were calculated from data or extrapolated from survival curves in the eligible studies; the HR information obtained by statistical software unavoidably developed a decrease of reliability. Fourth, all the included articles were retrospective studies; ideally, prospective studies would be required to generate more robust conclusions. In addition, since there are multiple sources of human SALP, the prognostic value of total SALP for osteosarcoma is limited by its lack of specificity [58]. Most studies did not exclude those patients with other diseases that also cause SALP levels elevation, which also decreased the precision of results.

SALP is a routine diagnostic test in clinical laboratories; measurement of SALP is simple, rapid, and cost-effective and provides valuable information for patients with osteosarcoma. In spite of the limitations mentioned above, our meta-analysis permits the conclusion that high SALP is obviously associated with poor OS or EFS and presence of metastasis when diagnosed. SALP level is a convenient and effective biomarker of prognosis for osteosarcoma.

## Figures and Tables

**Table 1:** Main characteristics and results of the eligible studies for evaluation of overall survival.

Study (author/year)	Country	Number of patients	Age (y) Median (range)	ALP cut-off (IU/L)	Enneking stage	HR (95% CI)	Follow-up (m) Median (range)
Aparicio et al., 1999 [25]	Spain	33	17 (12–42)	115	II	1.26 (0.42–3.74)	96 (60–156)
Foukas et al., 2002 [26]	UK	45	18 (6–48)	NA	IIB	3.44 (1.52–7.79)	68 (28–88)
Ford et al., 2004 [27]	UK	350	NS (<40)	NA	II	1.66 (1.09–2.51)	NA
Bramer et al., 2005 [28]	UK	89	NA	NA	II	1.49 (0.72–3.06)	NA
Mialou et al., 2005 [29]	France	60	13.5 (2–19)	500	IIIB	2.2 (1.2–4.1)	NA
Yalçın et al., 2008 [30]	Turkey	55	13 (7–17)	NA	II-III	1.1 (0.5–2.41)	NA
Chou et al., 2009 [31]	USA	91	NA	NA	III	3.16 (1.77–5.36)	89 (1–141)
Kim et al., 2009 [32]	Korea	67	15.7 (3.8–644)	NA	II	1.37 (0.54–3.45)	59.9
Wu et al., 2009 [33]	Taiwan ROC	91	20.2 (5–84)	A^†^	II-III	2.52 (1.32–4.75)	58.2 (2–233)
Yao et al., 2009 [34]	China	57	16 (6–70)	136	II-III	3.45 (1.4–8.46)	32.5 (10–52)
Hagleitner et al., 2011 [35]	Netherlands	94	17.8 (4.5–39.5)	NA	II-III	1.66 (0.88–3.11)	67.2 (28.8–360)
Ferrari et al., 2012 [36]	Italy	209	14 (4–39)	A^†^	II	1.69 (0.98–2.9)	76 (31–115)
Han et al., 2012 [37]	China	177	23.2 (5–57)	A^§^	II	1.80 (1.28–2.51)	87 (8–144)
Durnali et al., 2013 [38]	Turkey	211	20 (13–74)	A^$^	II-III	1.75 (1.23–2.5)	30.5 (0.5–213)
Min et al., 2013 [39]	China	333	19 (5–78)	NA	II-III	1.73 (1.28–2.33)	NA (1–100)
Hung et al., 2015 [40]	Taiwan ROC	69	13.5 (3.8–17.7)	150	II-III	3.08 (1.05–9.08)	51.6 (18–111.6)
Berner et al., 2015 [41]	Norway	301	NA	A^Δ^	II-III	1.64 (1.22–2.19)	NA

NA: not available, A: available (see the footnotes for details), HR: hazard ratio, CI: confidence interval, y: year(s), and m: month(s).

$: 2 times of upper limit normal level.

†: ALP cut-off: 2–10 y 100–350 IU/L; 10–13 y female 110–400 IU/L; 13–15 y male 125–500 IU/L; 20–50 y 25–100 IU/L; other childhood age 73–300 IU/L.

§: ALP cut-off: >18 y 150 IU/L; <18 y 110 IU/L.

Δ: ALP cut-off: 0–17 y 400 IU/L; >17 y 105 IU/L.

**Table 2:** Main characteristics and results of the eligible studies for evaluation of event-free survival.

Study (author/year)	Country	Number of patients	Age (y)	ALP cut-off (IU/L)	Enneking stage	HR (95% CI)	Follow-up (m) Median (range)
Mialou et al., 2005 [29]	France	48	13.5 (2–19)	500	IIIB	2.7 (1.5–4.8)	NA
Lee et al., 2007 [42]	Korea	45	<15	A^†^	II	4.55 (1.22–16.99)	54 (6–153)
Yalçın et al., 2008 [30]	Turkey	55	13 (7–17)	NA	II-III	1.11 (0.54–2.31)	NA
Kim et al., 2009 [32]	Korea	67	15.7 (3.8–64.4)	NA	II	1.84 (0.84–4.07)	59.9
Chou et al., 2009 [31]	USA	91	NA	NA	III	2.83 (1.68–4.79)	89 (1–141)
Ferrari et al., 2012 [36]	Italy	209	14 (4–39)	A^§^	II	1.71 (1.09–2.67)	76 (31–115)
Berner et al., 2015 [41]	Norway	237	NA	A^Δ^	II-III	1.78 (1.26–2.52)	NA

NA: not available, A: available (see the footnotes for details), HR: hazard ratio, CI: confidence interval, y: year(s), and m: month(s).

†: ALP cut-off: 2–10 y 420 IU/L; 10-11 y 560 IU/L; 12–15 y male 495 IU/L; 12-13 y female 420 IU/L; 14-15 y female 230 IU/L.

§: ALP cut-off: 2–10 y 350 IU/L; 10–13 y female 400 IU/L; 13–15 y male 500 IU/L; 20–50 y 100 IU/L; other childhood age 300 IU/L.

Δ: ALP cut-off: 0–17 y 400 IU/L; >17 y 105 IU/L.

**Table 3:** Main characteristics and results of the eligible studies for evaluation of presence of metastasis at diagnosis.

Study (author/year)	Country	Number of patients	Age (y)	ALP cut-off (IU/L)	Enneking stage	RR (95% CI)
Bacci et al., 1993 [43]	Italy	549	NA	A^§^	II-III	5.53 (2.85–10.74)
Wu et al., 2009 [33]	Taiwan ROC	91	20.2 (5–84)	A^§^	II-III	16.5 (1.05–260.27)
Hung et al., 2015 [40]	Taiwan ROC	76	13.5 (3.8–17.7)	150	II-III	18.6 (1.17–294.97)

NA: not available, A: available (see the footnotes for details), RR: relative risk, and CI: confidence interval.

§: ALP cut-off: 2–10 y 350 IU/L; 10–13 y female 400 IU/L; 13–15 y male 500 IU/L; 20–50 y 100 IU/L; other childhood age 300 IU/L.

**Table 4:** Main characteristics and results of the eligible studies for evaluation of metastasis development for nonmetastatic patients.

Study (author/year)	Country	Number of patients	Age (y)	ALP cut-off (IU/L)	Enneking stage	RR (95% CI)	Follow-up (m) Median (range)
Smeland et al., 2003 [44]	Norway	104	NA	A^†^	II	1.66 (0.68–4.07)	83 (42–124)
Han et al., 2012 [37]	China	177	23.2 (5–57)	A^§^	II	2.17 (0.97–4.84)	87 (8–144)
Kim et al., 2014 [45]	Korea	91	NA	A^§^	IIB	2.03 (1.04–3.97)	NA

NA: not available, A: available (see the footnotes for details), RR: relative risk, y: year(s), and m: month(s).

†: ALP cut-off: 2–10 y 350 IU/L; 10–13 y female 400 IU/L; 13–15 y male 500 IU/L; 20–50 y 100 IU/L; other childhood age 300 IU/L.

§: ALP cut-off: >14 y 115.5 IU/L; <14 y 300 IU/L.

**Table 5:** A summary of HRs for the overall and subgroup analyses of SALP and OS of osteosarcoma patients.

	Number of studies	Patients number	HR (95% CI)	Heterogeneity		
				Chi-squared	*I* ^2^	*p* value
Overall	17	2272	1.82 (1.61–2.06)	13.68	0%	0.622
Age						
Preadult and adult	14	2088	1.82 (1.60–2.06)	10.83	0%	0.625
Preadult only	3	184	1.87 (1.20–2.91)	2.84	2.96%	0.242
Enneking stage						
II	7	910	1.75 (1.42–2.15)	3.53	0%	0.740
II-III	8	1211	1.77 (1.61–2.06)	6.02	0%	0.538
III	2	151	2.67 (1.75–4.06)	0.71	0%	0.400
Sample size						
<100	11	751	2.13 (1.70–2.66)	10.94	8.6%	0.362
>100	6	1521	1.71 (1.48–1.98)	0.20	0%	0.999
Geographic region						
Asia	6	734	1.89 (1.55–2.31)	4.16	0%	0.527
Non-Asia	11	1538	1.78 (1.53–2.07)	9.29	0%	0.505

HR: hazard ratio, OS: overall survival, and CI: confidence interval.

**Table 6:** A summary of HRs for the overall and subgroup analyses of SALP and EFS of osteosarcoma patients.

	Number of studies	Patients number	HR (95% CI)	Heterogeneity		
				Chi-squared	*I* ^2^	*p* value
Overall	7	752	1.97 (1.61–2.42)	7.65	21.6%	0.265
Age						
Preadult and adult	4	385	1.90 (1.45–2.48)	5.33	43.7%	0.149
Preadult only	3	367	2.07 (1.51–2.83)	2.15	7.1%	0.341
Enneking stage						
II	3	321	1.88 (1.29–2.73)	1.91	0%	0.386
II-III	2	292	1.63 (1.19–2.23)	1.32	24.3%	0.250
III	2	139	2.77 (1.88–4.09)	0.01	0%	0.906
Sample size						
<100	5	306	2.28 (1.68–3.09)	6.09	34.3%	0.193
>100	2	446	1.75 (1.33–2.31)	0.02	0%	0.89
Geographic region						
Asia	2	112	2.34 (1.19–4.60)	1.34	25.2%	0.248
Non-Asia	5	640	1.94 (1.56–2.40)	6.05	33.8%	0.196

HR: Hazard ratio, EFS: event-free survival, and CI: confidence interval.
